# Immune Responses Against SARS-CoV-2 WT and Delta Variant in Elderly BNT162b2 Vaccinees

**DOI:** 10.3389/fimmu.2022.868361

**Published:** 2022-06-27

**Authors:** Michael Jäger, Sissy Therese Sonnleitner, Stefanie Dichtl, Eliott Lafon, Gabriel Diem, Gernot Walder, Cornelia Lass-Flörl, Doris Wilflingseder, Wilfried Posch

**Affiliations:** ^1^Institute of Hygiene and Medical Microbiology, Medical University of Innsbruck, Innsbruck, Austria; ^2^Medical Laboratory, Department of Virology, Dr. Gernot Walder GmbH, Ausservillgraten, Austria

**Keywords:** Age-dependent immune responses after BNT162b2, SARS-CoV-2 vaccination, T cell immunity, neutralizing antibodies, SARS-CoV-2, variants of concern (VOCs), COVID-19, virus neutralization

## Abstract

**Background:**

Residents of nursing homes are one of the most vulnerable groups during the severe acute syndrome coronavirus 2 (SARS-CoV-2) pandemic. The aim of this study was to characterize cellular and humoral immune responses in >70-year-old participants before vaccination, after first and second vaccination with BNT162b2, in contrast to second-dose-vaccinated participants younger than 60 years.

**Methods:**

Peripheral blood mononuclear cells of 45 elderly and 40 younger vaccinees were analyzed by IFNγ ELISpot, specific immunoglobulin G antibody titers against SARS-CoV-2 spike protein, and neutralization abilities against SARS-CoV-2 wild-type (WT) and Delta variant (B.1.617.2).

**Results:**

Our results clearly demonstrate a significantly increased T cell response, IgG titers, and neutralization activities against SARS-CoV-2 WT and Delta between first and second vaccination with BNT162b2 in elderly vaccinees, thereby highlighting the importance of the second booster. Interestingly, similar cellular and humoral immune responses against SARS-CoV-2 WT and Delta were found after the second vaccine dose in the young and elderly groups.

**Conclusions:**

Our data demonstrate a full picture of cellular and humoral immune responses of BNT162b2-vaccinees in two age cohorts. In all vaccines, SARS-CoV-2 WT-specific antibodies with similar neutralizing activity were detected in all vaccinees. After the second vaccination, neutralization titers against SARS-CoV-2 Delta were impaired in both age groups compared with SARS-CoV-2 WT, thereby emphasizing the need for an additional booster to overcome rising variants of SARS-CoV-2.

## Introduction

Since the end of 2020, COVID-19 vaccines have been authorized for use in the European Union. In particular, two messenger RNA (mRNA)-based COVID-19 vaccines, BNT162b2 (Comirnaty) from Biontech/Pfizer and mRNA-1273 from Moderna, are widely used in the Western world. In Austria and other European countries, COVID-19 vaccines were first offered to individuals ≥80 years of age with a high risk of severe or critical COVID-19 disease progression (1, 2). Since the Austrian vaccination committee recommends the use of mRNA vaccine BNT162b2 for the immunization of 65-year-olds and above, all people from the above-mentioned high-risk group and healthcare professionals were vaccinated with BNT162b2 at the start of the vaccination campaign in Austria.

The convincing efficacy of the vaccine BNT162b2 has been shown in various studies, primarily investigating immunogenicity in adults up to 65 years of age (3–5). Results from two-dose BNT162b2 vaccine studies of patients above 80 years of age are rare and show an age-dependent pattern in efficacy with considerable decreases in both humoral and cellular immune responses (6–8). Particularly with regard to the immune escape variants, age-related immune heterogeneity requires detailed examination. First studies proclaimed lower neutralization potency against the B.1.1.7 (Alpha), B.1.351 (Beta), and P.1. (Gamma) variants of concern (VOC) in middle and aged adults (7) and Delta (B.1.617.2) in young healthcare workers compared with the wild-type (WT) virus (9). Yet, the data availability concerning the immune competence and neutralization ability of aged BNT162b2 vaccinees is still scarce.

Therefore, in this study, we investigated the immunogenicity in elderly adults (>70 years of age) before, after the first and second doses of the BNT162b2 COVID vaccine and younger vaccines (<60 years old) after the second dose using IgG titers, neutralization tests and T cell response as diagnostic tools and are among the first to prove its efficacy against the B.1.617.2 (Delta) VOC in the elderly.

## Methods

### Ethics Statement

All participants were informed about the study in the course of the medical consultation before vaccination, provided written informed consent, and the study was performed according to the principles of the declaration of Helsinki 2013. The Ethics Committee of the Medical University of Innsbruck approved the use of anonymized leftover specimens of COVID-19 patients or vaccinees (ECS1166/2020) and healthy donors (ECS1166/2018) for scientific purposes.

### Human Samples

In this study, 89 people were included and divided into two groups: A (**Table 1**) covers people older than 70 years (n = 45; average age 83 years (70–94 years)) and B (**Table 2**) includes adults younger than 60 years (n = 40; average age 44 years (22–61 years)). All participants were fully vaccinated with the BNT162b2 vaccine. None of the enrolled participants had a recent, current, or persistent infectious disease or treatment with steroids and/or immunosuppressants. The percentage of females was 51% for people over 70 year old and 48% for persons younger than 60 years, respectively. For aged participants (group A), the average sampling day after the 1st vaccination was 22 (days 20–28) and 37 (days 34–49) after the 2nd dose, respectively. For people of middle age (group B), the average day of sampling after full vaccination was 35 (days 15–78). Detailed information of each individual from the two groups regarding age, sex, time after first and second vaccinations, as well as inclusion in neutralization and T cell analysis is presented in **Tables 1** and **2**.

**Table 1:** Characteristics from individuals older than 70 years vaccinated with BNT162b2 included in the study (n = 45).

ID	Age	Gender	Days after 1st dose	Days after 2nd dose
A1	81	m	28	49
A2	81	f	28	49
A3	86	f	28	49
A4	74	m	28	49
A5	83	m	28	49
A6	85	f	28	49
A7	83	f	28	49
A8	89	f	28	40
A9	88	m	20	34
A10	86	m	20	34
A11	84	f	20	34
A12	89	f	20	34
A13	74	f	20	34
A14	83	m	20	34
A15	94	m	20	34
A16	83	f	20	34
A17	81	m	20	34
A18	85	f	20	34
A19	86	m	20	34
A20	85	m	20	34
A21	89	f	20	34
A22	92	m	20	34
A23	89	m	20	34
A24	86	f	20	34
A25	82	f	20	34
A26	81	f	20	34
A27	91	m	20	34
A28	85	f	20	34
A29	81	m	20	34
A30	80	m	20	34
A31	80	f	20	34
A32	88	m	20	34
A33	78	f	20	34
A34	71	f	20	34
A35	84	m	20	34
A36	87	m	20	34
A37	75	f	20	37
A38	70	f	20	34
A39	83	f	21	35
A40	91	f	21	35
A41	79	f	21	35
A42	81	m	21	35
A43	88	m	21	35
A44	83	m	21	35
A45	79	m	21	35
**Median age/years**	**83**			
**n (female) / %**	**51**			
**Median days after 1st dose**	**20**			
**Median days after 2nd dose**	**34**			

Age, sex, and sampling day after the first and second vaccine dose are shown. The average age of the participants was 83 years. The percentage of women was 51%. Samples of BNT162b2 vaccinees older than 70 years were taken between 20 and 28 days after the first dose and between 34 and 49 days after the second dose, respectively.

**Table 2:** Characteristics from individuals younger than 60 years vaccinated with BNT162b2 included in the study (n = 40).

ID	Age	Gender	Days after 2nd dose	NT analysis	T cell analysis
B1	48	f	34	yes	yes
B2	58	f	35	yes	yes
B3	58	m	35	yes	yes
B4	45	f	15	yes	yes
B5	51	f	22	yes	yes
B6	51	f	21	yes	yes
B7	52	m	21	yes	yes
B8	57	m	22	yes	yes
B9	58	m	22	yes	yes
B10	61	f	78	yes	yes
B11	30	m	78	yes	yes
B12	30	m	78	yes	yes
B13	26	f	78	yes	yes
B14	42	f	78	yes	yes
B15	33	m	78	yes	yes
B16	33	m	78	yes	yes
B17	22	m	78	yes	yes
B18	36	f	21	no	yes
B19	57	m	33	no	yes
B20	35	m	40	no	yes
B21	57	f	22	yes	no
B22	41	m	22	yes	no
B23	51	m	22	yes	no
B24	42	f	22	yes	no
B25	58	m	22	yes	no
B26	35	f	22	yes	no
B27	25	m	22	yes	no
B28	30	f	22	yes	no
B29	35	m	22	yes	no
B30	35	f	22	yes	no
B31	58	m	22	yes	no
B32	33	f	22	yes	no
B33	61	f	22	yes	no
B34	34	f	22	yes	no
B35	44	m	22	yes	no
B36	50	m	22	no	no
B37	53	f	22	no	no
B38	33	m	22	no	no
B39	52	f	22	no	no
B40	50	m	22	no	no
**Median age/years**	**45**				
**n (female) / %**	**48**				
**Median days after 2nd dose**	**22**				

Age, sex, and sampling day after the second vaccine dose are shown. Serum samples used for the neutralization experiments are labeled in column ‘NT analysis.’ PBMCs used for ELISpot assays are labeled in column ‘T cell analysis.’ The average age of the participants was 44 years. The percentage of women was 48%. All samples of BNT162b2 vaccinees younger than 60 years were taken between 15 and 78 days after the second dose.

### Viruses

The clinical specimen for SARS-CoV-2 (Delta; B.1.617.2) from COVID-19 positive swab (Ethics statement, ECS1166/2020) and SARS-CoV-2 virus (WT) from repositories (BEI Resources, Manassas, VA, USA; CFAR/NIBSC; Nr-52281) was propagated according to the instructions of the manufacturer and used subsequently in neutralization assays.

### Chemiluminescent Immunoassay (CLIA)

The LIAISON^®^ SARS-CoV-2 TrimericS IgG is a CLIA (Chemiluminescent Immunoassay) that detects IgG antibodies reactive with the spike protein (S1/S2 domain). The assay was performed according to the instructions of the manufacturer and gives the arbitrary units per ml (AU/ml) according to the WHO International Standards for the Anti-SARS-CoV-2 immunoglobulin binding activity (NIBSC 20-136).

### Neutralization Plaque Assay

VeroE6/TMPRSS2 cells (1.2 × 10^5^) were cultured as described previously (10). On the following day, whole serum or plasma samples after the first dose were serial-diluted from 1:4 to 1:512, while samples taken after the second dose were serial-diluted from 1:8 to 1:1,024. Dilutions were incubated with SARS-CoV-2 wild type or variant strains (4 × 10^2^ PFU·ml^−1^) for 1 h at 37°C. After incubation, VeroE6/TMPRSS2 cells were inoculated with antibody-opsonized SARS-CoV-2 for 1 h at 37°C with 5% CO_2_. After incubation, the inoculum was replaced with culture medium containing 1.5% carboxymethylcellulose (Sigma Aldrich). Cells were incubated for 3 days at 37°C and 5% CO_2_ before plaque visualization and counting. For this, cells were washed, fixed, and stained using 0.5% (w/v) Crystal Violet solution (Sigma Aldrich). To determine NT50 and NT90 values from neutralization curves, four-parameter nonlinear regression in GraphPad Prism v.9 was used.

### SARS-CoV-2-Specific T Cell Response

The ELISpot assay was performed using precoated human SARS-CoV-2-specific IFNγ ELISPOT kits (AutoImmun Diagnostika). Peripheral blood was collected, PBMCs were isolated and erythrocytes depleted (RBD-Lyse Buffer Life Technologies). PBMCs were counted and resuspended in X-VIVO medium (X-VIVO TM-10 Serum-free Hematopoietic Cell Medium, Lonza).

In brief, a total of 2 × 10^5^ PBMCs were incubated in duplicates with X-VIVO as a negative control, pokeweed mitogen (AutoImmun Diagnostika GmbH) as a positive control, and 15–20mer peptide pools for SARS-CoV-2 and PanCorona for the four non-SARS human coronaviruses 229E, HKU1, NL63, and OC43 (AutoImmun Diagnostika GmbH) as a control of possible cellular cross-reactive responses. After incubation at 37°C and 5% CO_2_ for 20 h, plates were washed with washing buffer (AutoImmun Diagnostika GmbH) and stained with the kit-specific reagents according to the protocol of the manufacturer. Spots were counted using an automated AID ELISPOT reader system (AutoImmun Diagnostika GmbH).

The stimulation index (SI) was calculated by dividing the mean spot numbers in the antigen-specific wells with the mean spot numbers of the negative control. A test was assessed as negative with an SI <2 and positive with an SI ≥2.

### Statistical Analysis

Statistical analysis was performed using GraphPad Prism v.9. The statistical significance of SARS-CoV-2-specific antibody titers, T-cell responses, and NT50 and NT90 levels between values of the 3 different time points in the group >70 y.o. was determined using the Wilcoxon test (GraphPad Prism). A Mann–Whitney–U test for nonparametric distribution was applied for comparison of immune responses between groups >70 y.o. and <60 y.o. (GraphPad Prism). The correlation of SARS-CoV-2 Spike T cell response vs. anti-SARS−CoV−2 S IgG as well as anti-SARS−CoV−2 S IgG vs. NT50 WT or Delta variant for participants older than 70 years or younger than 60 years was determined using two-tailed non-parametric Spearman correlation analyses (GraphPad Prism).

## Results

### T Cell response Against SARS-CoV-2 Is Age-Independent

**Figure 1:**
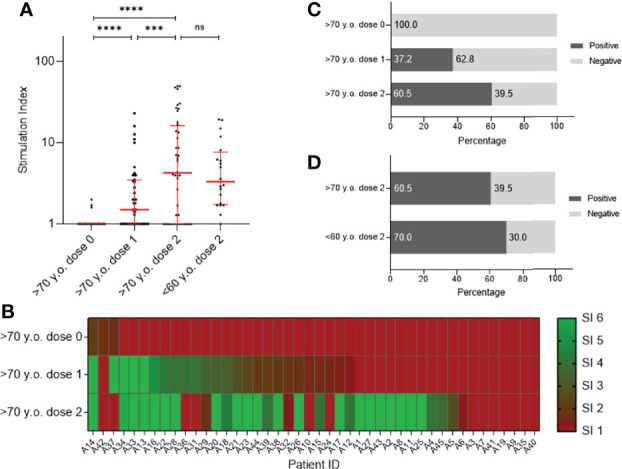
Analysis of T cells against SARS-CoV-2 spike using sera from participants older than 70 years (n = 45) before vaccination (dose 0), after first- (dose 1) and after second vaccination (dose 2) with BNT162b2 in contrast to fully vaccinated participants younger than 60 years (n = 20). The ELISpot assays were performed using precoated human SARS-CoV-2-specific IFNγ ELISPOT kits. Results are presented as a Stimulation Index (SI). **(A)** T cell responses against SARS-CoV-2 spike are shown as individual progression for each person (>70 y.o.) until full immunization with BNT162b2. In contrast, individual SARS-CoV-2 T cell responses are illustrated for people younger than 60 years of age fully vaccinated with BNT162b2. Medians are visualized together with the interquartile range as an error bar. Statistical significance between values of the three different time points for the group >70 y.o. was determined using the Wilcoxon test. A Mann–Whitney–U test for nonparametric distribution was applied for comparison of immune responses between the two age groups. [p > 0.05 not significant (ns), p ≤ 0.001 (***) and p≤ 0.0001 (****)]. **(B)** Heat map visualizing individual progression of T cell response against SARS-CoV-2 spike before and after vaccination with BNT162b2. Illustrated colors shift from red for a negative to green for a positive antibody status. **(C)** Percentages of participants with positive or negative T cell response against SARS-CoV-2 spike is shown for time points before-, after the first- and second vaccination. **(D)** Comparison of percentages of persons older than 70 years and people younger than 60 years with positive or negative T cell response against SARS-CoV-2 Spike after second vaccination.

To monitor SARS-CoV-2 specific T cells in participants older than 70 years (n = 45; **Table 1**) before vaccination, after first and second vaccination with BNT162b2, in contrast to second-dose vaccinated participants younger than 60 years (n = 20; **Table 2**), we collected peripheral blood and analyzed SARS-CoV-2-specific T cell response against the SARS-CoV-2 spike protein. The average age of the elderly group was 83 years, in comparison to the younger participants, who were on average 44 years old. Peripheral blood mononuclear cells (PBMCs) were stimulated with peptide pools to assess the functionality of SARS-CoV-2 spike-specific T cell numbers within the various groups. After 20 h, IFNγ spots were counted, and stimulation indices (SI) were calculated and used as a parameter for the analyzed T cell response against SARS-CoV-2. **Figure 1A** shows COVID-19-naive participants older than 70 years before vaccination (>70 y.o. dose 0), after first (>70 y.o. dose 1) and second vaccination (>70 y.o. dose 2), in contrast to second-dose vaccinated participants younger than 60 years (<60 y.o. dose 2). The median SI for detection of virus-specific T cells was found to be SI = 1.0 (95% CI, 1.0–1.0), SI = 1.5 (95% CI, 1.0–2.0) and SI = 4.3 (95% CI, 1.7–8.7) for individuals in the elderly group before, after the first and after the second dose of BNT162b2, respectively (**Figure 1A**; **Supplementary Table 1** upper panel). These analyses demonstrate the induction spike-specific T cell numbers after the first vaccination and that they significantly increased between the first and second vaccination in participants older than 70 years. The T cell response against SARS-CoV-2 were lower in the younger group of BNT162b2 vaccinees and showed a SI of 3.3 (95% CI, 1.8–6.2; **Figure 1A** and **Supplementary Table 1 upper part**). However, there was no age-dependent significant difference detected between second dose-vaccinated young (<60 y.o. dose 2) or elderly (>70 y.o. dose 2) participants (**Figure 1A**). **Figure 1B** additionally displays the individual progression of SARS-CoV-2-spike-specific T cells before and after vaccination with BNT162b2 in people >70 years old as a heatmap. The percentages of participants older than 70 years with positive or negative T cell response against SARS-CoV-2 Spike protein before vaccination, after first and second vaccination are summarized in **Figure 1C**. After the first vaccine dose, 37.2% were identified as positive for SARS-CoV-2-specific T cells, which was enhanced due to the second vaccine dose and reached a value of 60.5% (**Figure 1C**). In comparison, 70.0% of individuals from the younger group demonstrated virus-specific T cells, which demonstrates a 9.5% increase of individuals with specific T cell immunity compared to aged vaccinees. Nonetheless, 39.5% of individuals from the elderly and 30% from the younger group showed no detectable SARS-CoV-2-specific T cell response after the second vaccine dose (**Figure 1D**). These results show that when using the mRNA-based vaccine BNT162b2 in elderly vaccinees, the second dose is essential in order to obtain a comparable, but still lower virus-specific T cell response than younger participants.

### High SARS-CoV-2-Specific IgG Antibody Titers in Young and Older Subjects

**Figure 2:**
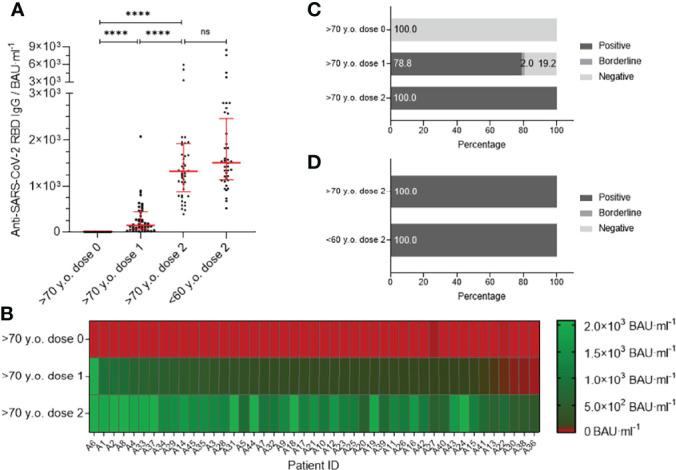
SARS-CoV-2 antibody titer analysis using sera from participants older than 70 years (n = 45) before vaccination (dose 0), after first- (dose 1) and after second vaccination (dose 2) with BNT162b2 in contrast to second dose vaccinated participants younger than 60 years of age (n = 40). Experiments for participants were executed with chemiluminescent immunoassay DiaSorin Trimeric S^®^ IgG against SARS-CoV-2-S. Results are presented in binding antibody units per ml of serum (BAU·ml^−1^). **(A)** IgG antibody titers against SARS-CoV-2 S domain are shown as individual progression for each person until full immunization with BNT162b2. In contrast, individual anti-SARS-CoV-2 IgG titers are illustrated for people younger than 60 years fully vaccinated with BNT162b2. Medians are visualized together with the interquartile range as an error bar. Statistical significance between values of the three different time points for the group >70 y.o. was determined using the Wilcoxon test. A Mann–Whitney–U test for nonparametric distribution was applied for comparison of immune responses between the two age groups. [p > 0.05 not significant (ns), and p ≤ 0.0001 (****)]. **(B)** Heat map for visualization of individual IgG antibody titer progression against SARS-CoV-2 S domain before and after vaccination with BNT162b2. Illustrated colors shift from red for a negative to green for a positive antibody status. **(C)** Percentages of participants positive, borderline or negative for IgG antibodies against SARS-CoV-2 S1 are shown for time points before-, after the first and second vaccination. **(D)** Comparison of percentages of persons older than 70 years and people younger than 60 years positive, borderline or negative for IgG antibodies against SARS-CoV-2 S.

Using ELISA-based testing methods, we investigated the presence of SARS-CoV-2-specific IgG antibodies against the S region of the SARS-CoV-2 spike protein in serum samples. **Figure 2A** demonstrates the levels of IgG antibodies against the S region of the SARS-CoV-2 spike protein before vaccination (4.8; 95% CI 4.8–4.8), after the first (142.0; 95% CI 107.0–260.0) and second (1,330.0; 95% CI 1,120.0–1,600.0) vaccine dose of participants >70 years old. The IgG antibodies were dramatically increased in elderly vaccines after the second dose of BNT162.b2 and reached a comparable amount to the younger population (1,512.0; 95% CI 1,291.0–1,827.0), which was twice vaccinated (**Figure 2A**; **Supplementary Table 1**, middle part). The individual progression of IgG antibodies before and after vaccination with BNT162b2 in older people is shown in a heatmap in **Figure 2B**. After the first vaccine dose, 78.8% of the participants >70 years old already had a positive IgG antibody response (**Figure 2C**), which was further increased to 100% after the second dose and therefore identical to the results of the younger population (100%) (**Figure 2D**). The results highlight that the IgG antibody response is equivalent between the two age cohorts after the second dose of BNT162b2.

### Elderly Vaccinees Show Comparable SARS-CoV-2 WT Neutralizing Capacity Compared With Younger Participants

**Figure 3:**
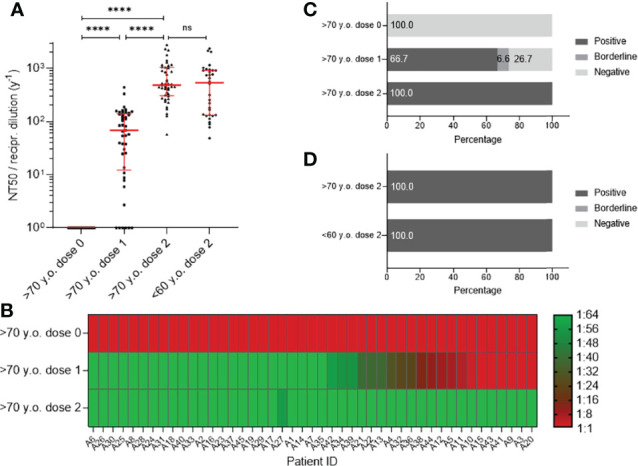
Analysis of neutralization titer (NT50) against SARS-CoV-2 WT using sera from participants older than 70 years (n = 45) before vaccination (dose 0), after first- (dose 1), and after second vaccination (dose 2) with BNT162b2, in contrast to fully vaccinated participants younger than 60 years (n = 32). Neutralization titers are presented as a dilution factor for serum (1:x). **(A)** Individual NT50 values are shown as individual progressions for each person until full immunization with BNT162b2. In contrast, individual titers for 50% neutralization are illustrated for people younger than 60 years of age fully vaccinated with BNT162b2. Medians are visualized together with the interquartile range as an error bar. Statistical significance between values of the three different time points for the group >70 y.o. was determined using the Wilcoxon test. A Mann–Whitney–U test for nonparametric distribution was applied for comparison of immune responses between the two age groups. [p > 0.05 not significant (ns), and p ≤ 0.0001 (****)]. **(B)** Heat map for visualization of NT50 progression against SARS-CoV-2 WT before and after vaccination with BNT162b2. Illustrated colors shift from red for a negative- to green for a positive antibody status. **(C)** Percentages of participants with positive, borderline or negative for half-maximum neutralization against SARS-CoV-2 WT are shown for time points before-, after the first and second vaccination. **(D)** Comparison of percentages of persons older than 70 years and people younger than 60 years with positive, borderline or negative half-maximum neutralization of SARS-CoV-2 WT virus.

To further investigate the differences after vaccination between the >70-year-old and <60-year-old populations, we determined the virus neutralization abilities of these two groups by analyzing half maximal neutralization titers (NT50, **Figure 3**) and neutralization titers that inhibit 90% of viral infection (NT90, **Supplementary Figure 1**). In this regard, serum samples were incubated at various concentrations with SARS-CoV-2 WT. Analysis of the NT50 values of participants >70 years revealed that between the first and the second vaccination, virus neutralization abilities significantly increased (68.1; 95% Cl 37.8–119.6) and resulted in comparable NT50 values between the two age cohorts after the second vaccine (>70 years old: 481.8; 95% Cl 415.7–768; <60 years old: 536; 95% Cl 134.3–909.4) (**Figure 3A**; **Supplementary Table 1**, lower part). These results were also confirmed for the NT90 values (**Supplementary Figure 1A**). The individual progression of NT50 (**Figure 3B**) and NT90 (**Supplementary Figure 1B**) values before and after first and second vaccination with BNT162b2 in the elderly vaccinees is shown as a heatmap. We further analyzed the data and found that 66.7% or 28.9% of >70-year-old individuals demonstrated positive NT50 or NT90 values against SARS-CoV-2 WT, respectively (**Figure 3C**; **Supplementary Figure 1C**). Positive neutralization of NT50 after the second vaccination was detected in all individuals of both age groups (100%, **Figure 3D**). Additionally, 88.9% of the aged participants showed positive NT90 neutralization, which was lower than the younger group (93.8%) (**Supplementary Figure 1D**).

### Virus Neutralization Abilities Against SARS-CoV-2 Delta Variant

**Figure 4:**
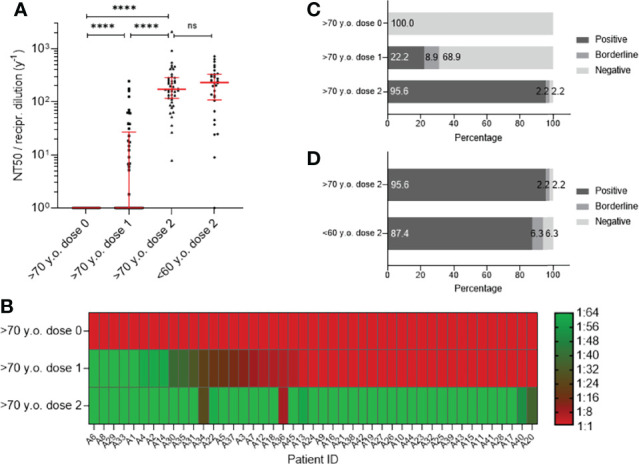
Analysis of neutralization titer (NT50) against SARS-CoV-2 Delta variant using sera from participants older than 70 years (n = 45) before vaccination (dose 0), after first- (dose 1) and after second vaccination (dose 2) with BNT162b2 in contrast to fully vaccinated participants younger than 60 years (n = 32). Neutralization titers are presented as a dilution factor for serum (1:x). **(A)** Individual NT50 values are shown as individual progressions for each person until full immunization with BNT162b2. In contrast, individual titers for 50% neutralization are illustrated for people younger than 60 years of age fully vaccinated with BNT162b2. Medians are visualized together with the interquartile range as an error bar. Statistical significance between values at the three different time points for the group >70 y.o. was determined using the Wilcoxon test. A Mann–Whitney–U test for nonparametric distribution was applied for comparison of immune responses between the two age groups. [p > 0.05 not significant (ns), and p ≤ 0.0001 (****)].. **(B)** Heat map for visualization of NT50 progression against the SARS-CoV-2 WT before and after vaccination with BNT162b2. Illustrated colors shift from red for a negative- to green for a positive antibody status. **(C)** Percentages of participants with positive, borderline or negative for half-maximum neutralization against SARS-CoV-2 Delta variant are shown for time points before-, after the first and second vaccination. **(D)** Comparison of percentages of persons older than 70 years and people younger than 60 years with positive, borderline or negative half-maximum neutralization of SARS-CoV-2 Delta virus.

At the time of analysis, the B.1.617.2 (Delta) variant was the dominant circulating variant worldwide, causing increasing severity of disease progression and more transmissions (11, 12). Therefore, we were interested in analyzing neutralization titers in this VOC of SARS-CoV-2. In general, neutralization titers against the Delta variant were impaired in both age cohorts compared to SARS-CoV-2 WT, and median NT50 values after the second vaccine dose decreased from 536.0 to 231.5 and from 481.8 to 172.6 for young and elderly participants, respectively (**Figures 4C, D**, **Supplementary Table 1, lower part**). Similar to SARS-CoV-2 WT, NT50 (**Figure 4A**) and NT90 (**Supplementary Figure 2A**) titers against SARS-CoV-2 Delta significantly increased between the first and second vaccine doses in participants >70 years old, similar to SARS-CoV-2 WT. After the second dose of BNT162b2, no significant difference in NT50 or NT90 values were detected between the two age cohorts (NT50 of >70 years old: 172.6; 95% Cl 137.2–240.5; NT50 of <60 years old: 231.5; 95% Cl 131.1–309.9; **Supplementary Table 1, lower part**). The individual progression of NT50 (**Figure 4B**) and NT90 (**Supplementary Figure 2B**) values against SARS-CoV-2 Delta before and after the first as well as after the second vaccination in the aged population is displayed as heatmaps. After the first vaccination, 22.2% of the participants >70 years old had positive neutralization titers (NT50) against the SARS-CoV-2 Delta variant (**Figure 4C**), which was further increased to 95.6% after the second dose and therefore comparable to the results of the younger population (87.4%) (**Figure 4D**). In contrast to NT50, NT90 was decreased and only 6.7% of the elderly vaccinees after the first vaccination and 75.6% after the second vaccination were positive (**Supplementary Figure 2C**). In the younger participants, 81.3% were positive after the second dose (**Supplementary Figure 2D**).

### Correlation Between IgG Antibody Titers, T Cell Response, and NT50 Values

**Figure 5:**
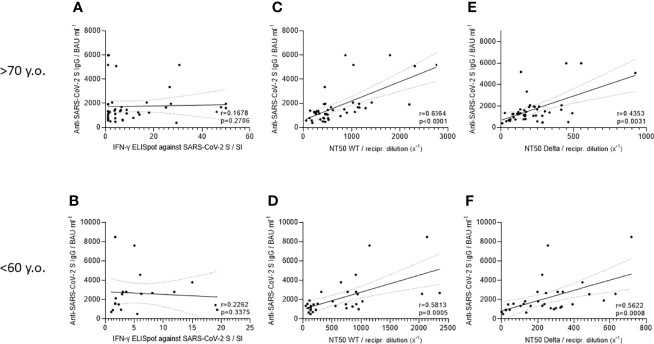
Correlation of T cell response against SARS-CoV-2 spike, anti-SARS-CoV-2 S IgG titer and NT50 values against WT and Delta variant for participants younger than 60 years or older than 70 years. Dependencies between T cell response against SARS-CoV-2 spike, SARS-CoV-2-specific IgG, and NT50 values from SARS-CoV-2 WT and Delta virus were calculated by nonparametric Spearman correlation analysis. Correlations for participants older than 70 years were visualized as **(A)** T cell response (IFNγ ELISpots) vs. IgG (n = 45), **(C)** IgG response vs. NT50 values against WT virus (n = 45) and **(E)** IgG vs. NT50 against Delta variant (n = 45). To improve visualization of the trend, a linear regression with a 95% confidence interval (CI) was plotted. SARS-CoV-2-specific T cells (IFNγ ELISpots) are presented as Stimulation Index (SI), IgG titers in binding antibody units per ml of serum (BAU·ml^−1^) and NT50 values as a dilution factor for serum (1:x). The same correlations were determined for people younger than 60 years **(B)** (n = 20), **(D)** (n = 32), and **(F)** (n = 32).

Results from IgG antibody titers, T cell responses, and neutralization assays (NT50 values) observed in double vaccinated people >70 and <60 years old were statistically analyzed to identify a correlation. First, we correlated IgG antibody titers and T cell responses against SARS-CoV-2 spike protein from each individual in the elderly (**Figure 5A**) and in the younger (**Figure 5B**) cohorts using two-tailed non-parametric Spearman correlation analyses. For IgG/T cell correlation, we could not detect any significant correlation in either age group. In comparison, the correlation of IgG antibody titers and NT50 values against SARS-CoV-2 WT was significantly positive in aged (r = 0.6325; p <0.0001) (**Figure 5C**) and young participants (r = 0.5925; p = 0.0004) (**Figure 5D**). Similar results were also obtained for the calculation of IgG/NT50 against the SARS-CoV-2 Delta variant. In fact, we found a significant positive correlation for >70-year-old people (r = 0.4645; p = 0.0013) (**Figure 5E**) and for <60-year-old vaccinees (r = 0.58; p = 0.0005) (**Figure 5F**).

### Sex-Related Effects of Humoral Immune Response and Virus Neutralization

Finally, we compared the humoral immune response and virus neutralization between females and males in >70- and <60-year-old individuals. Virus-specific T cells against SARS-CoV-2 Spike protein (**Supplementary Figure 3A**) and IgG antibody titers (**Supplementary Figure 3B**) were not influenced by sex or age. In contrast, NT50 values against SARS-CoV-2 WT significantly differed between females and males in the aged population (**Supplementary Figure 3C**). We observed increased NT50 values in elderly women (897.5; 95% CI 524.3–958.7) compared with elderly men (522.0; 95% CI 263.7–548.1). Furthermore, we could detect a decrease in NT50 values in <60-year-old women (530.1; 95% CI 167.4–561.2) compared to >70-year-old women. This age- and sex-dependent effect was not present in NT50 values against SARS-CoV-2 Delta (**Supplementary Figure 3D**).

## Discussion

The aims of this study were to analyze humoral immune response and neutralization titers in aged people (>70 years old) before, after the first and second vaccination with BNT162b2, and in a younger cohort (<60 years old) after their second vaccine dose. We further investigated the neutralization titers against SARS-CoV-2 WT and SARS-CoV-2 Delta. Previous studies evaluated the effects of vaccination regarding SARS-CoV-2 Delta in young people or investigated age-dependent effects after vaccination against SARS-CoV-2 WT, but there is, to our knowledge, no combination study so far. To study the humoral immune response, we analyzed the T cell response against SARS-CoV-2 spike protein and the titers of IgG antibodies. Studies showed that T cell response might limit disease progression when neutralization titers are low and IgG, among others, correlates with serum neutralization, which causes the dominant protection against SARS-CoV-2 infection (13). Our data show comparable stimulation indices and positivity of T cell responses between aged and younger participants, since no significant difference between both groups could be detected. These results are in agreement with the literature, which also showed an impaired T-cell response after two doses of BNT162b2 in older people (14). IgG antibody titers significantly increased between the first and second vaccination, which was previously demonstrated (6). Surprisingly, we were not able to detect significant differences in the IgG levels between the two age cohorts, despite the fact that the antibody titers of the younger vaccinees were higher. Although recent studies demonstrated an age-dependent decline in IgG antibodies after the second vaccination with BNT162b2 (6, 14), our results showed that the percentage of participants, who were positive for IgG antibodies did not differ between younger and older vaccinees. One reason for the observed differences could be the prolonged interval between the second vaccination and analysis, which was 60 days in the study of Demaret et al. and 37 days in our study (14). Another explanation for the detected differences between previous studies and the data presented here might be the age of the elderly population. Causa et al. showed that the age-related decline of IgG titers has been particularly appreciable among individuals aged 80 or more (15). In their study, Muller et al. (6) included aged people with a median age of 88 years (80.1–100.5 years), in contrast to our study, in which the average age was lower (70–94 years). This decreased median age of study participants and the inclusion of people starting from 70 years in our study display differences, which could be responsible for the observed age-independent effect for IgG (6).

Neutralizing antibodies are a surrogate parameter for the efficiency of vaccines. Our study demonstrates that the NT50 of both age cohorts is comparable after the second vaccination, which was also reported by Collier et al. (7). Our data further revealed a strong positive correlation between IgG antibody titers and NT50 values against SARS-CoV-2 WT in both age cohorts, which is consistent with recently published data (6). Analysis of sex-specific effects concerning the neutralizing antibodies after the second vaccination revealed that aged women had significantly higher NT50 levels against SARS-CoV-2 WT compared with elderly men. This is in accordance with other published data, which showed more neutralizing antibodies in female octogenarians after BNT162b2 vaccination (16). To our knowledge, this is the first report on sex-dependent analysis of neutralizing antibodies after immunization with BNT162b2 in an age-related comparison. Therefore, further studies with an increased number of participants are required to confirm our findings on sex-related vaccination efficacy.

In the case of the SARS-CoV-2 Delta variant, our data emphasized the importance of the second vaccination in the elderly. Interestingly, the percentages of positive neutralizing titers were even higher in >70-year-old participants (95.6%) compared to <60-year-old ones (87.4%). Additionally, we found that the percentage of younger twice-vaccinated participants with positive half-maximum neutralization was 12.6% decreased in the SARS-CoV-2 Delta variant in contrast to SARS-CoV-2 WT. This observation is supported by previous studies presenting a decreased protection of BNT162b2 against the SARS-CoV-2 Delta variant (9, 17). We also detected a significant positive correlation of IgG antibody titers and NT50 values against SARS-CoV-2 Delta in both age cohorts. Therefore, we concluded that the observed humoral responses were protective even against SARS-CoV-2 Delta. Further studies are needed to confirm this age-independent correlation in other SARS-CoV-2 variants as well.

Limitations of this study include the small number of study participants and the further assessment after the recommended third vaccination of BNT162b2. The relatively short follow-up period only allowed for short-term effects of the vaccine. Studies with the recently discovered SARS-CoV-2 Omicron variant, which is associated with lower COVID-19 vaccine effectiveness, are needed to analyze the vaccination efficiency in different age cohorts and sexes in order to protect the most vulnerable groups in our society (18).

## Data Availability Statement

The original contributions presented in the study are included in the article/**Supplementary Material**. Further inquiries can be directed to the corresponding author.

## Ethics Statement

The studies involving human participants were reviewed and approved by the Ethics Committee of the Medical University of Innsbruck (ECS1166/2020) (ECS1166/2018). The patients/participants provided their written informed consent to participate in this study.

## Author Contributions

Conceptualization, CL-F, DW, and WP. Methodology, MJ, SS, GW, DW, and WP. Validation, MJ, SS, EL, GD, and WP. Formal analysis, MJ, SS, SD, EL, GD, DW, and WP. Investigation, MJ, SS, SD, and WP. Resources, SS, GW, DW, CL-F, WP. Data curation, MJ, SS, SD, EL, GD, DW, and WP. Writing—original draft preparation, MJ, SS, SD, DW, and WP. Writing—review and editing, MJ, SS, SD, EL, GD, GW, CL-F, DW, and WP. Visualization, MJ and WP. Supervision, CL-F and WP. Project administration, WP. Funding acquisition, GW, CL-F, and WP. All authors listed have made a substantial, direct, and intellectual contribution to the work and approved it for publication.

## Funding

The authors were supported by the Austrian Science Fund (FWF; P34070-B13 to WP and P33510-B13 to DW), the Anniversary Fund of the Austrian National Bank (OeNB; P17614 to WP, P17633 to DW), and the State of Tyrol (No. 70454 to WP).

## Conflict of Interest

Authors SS and GW were employed by Dr. Gernot Walder GmbH, Ausservillgraten, Austria.

The remaining authors declare that the research was conducted in the absence of any commercial or financial relationships that could be construed as a potential conflict of interest.

## Publisher’s Note

All claims expressed in this article are solely those of the authors and do not necessarily represent those of their affiliated organizations, or those of the publisher, the editors and the reviewers. Any product that may be evaluated in this article, or claim that may be made by its manufacturer, is not guaranteed or endorsed by the publisher.

## Supplementary Material

Supplementary Figure 1 Analysis of neutralization titer (NT90) against SARS-CoV-2 WT using sera from participants older than 70 years (n=45) before vaccination (dose 0), after first- (dose 1) and after second vaccination (dose 2) with BNT162b2 in contrast to fully vaccinated participants younger than 60 years (n=32). Neutralization titers are presented as dilution factor for serum (1:x). **(A)** Individual NT90 values are shown as individual progression for each person until full immunization with BNT162b2. In contrast, individual titers for 90% neutralization are illustrated for people younger than 60 years fully vaccinated with BNT162b2. Medians are visualized es together with the interquartile range as error bar. Statistical significance between values of the three different time points for group >70 y.o. was determined using Wilcoxon test. Mann-Whitney-U test for nonparametric distribution was applied for comparison of immune responses between the two age groups. **(B)** Heat map for visualization of NT90 progression against SARS-CoV-2 WT before and after vaccination with BNT162b2. Illustrated colors shift from red for a negative- to green for a positive antibody status. **(C)** Percentages of participants with positive, borderline or negative for 90% neutralization against SARS-CoV-2 WT are shown for time points before-, after first- and after second vaccination. **(D)** Comparison of percentages of persons older than 70 years and people younger than 60 years with positive, borderline or negative 90% neutralization of SARS-CoV-2 WT virus.

Supplementary Figure 2 Analysis of neutralization titer (NT90) against SARS-CoV-2 Delta variant using sera from participants older than 70 years (n=45) before vaccination (dose 0), after first- (dose 1) and after second vaccination (dose 2) with BNT162b2 in contrast to participants younger than 60 years (n=32) fully vaccinated. Neutralization titers are presented as dilution factor for serum (1:x). **(A)** Individual NT90 values are shown as individual progression for each person until full immunization with BNT162b2. In contrast, individual titers for 90% neutralization are illustrated for people younger than 60 years fully vaccinated with BNT162b2. Medians are visualized together with the interquartile range as error bar. Statistical significance between values of the three different time points for group >70 y.o. was determined using Wilcoxon test. Mann-Whitney-U test for nonparametric distribution was applied for comparison of immune responses between the two age groups. **(B)** Heat map for visualization of NT90 progression against SARS-CoV-2 Delta variant before and after vaccination with BNT162b2. Illustrated colors shift from red for a negative- to green for a positive antibody status. **(C)** Percentages of participants with positive, borderline or negative for 90% neutralization against SARS-CoV-2 Delta variant are shown for time points before-, after first- and after second vaccination. **(D)** Comparison of percentages of persons older than 70 years and people younger than 60 years with positive, borderline or negative 90% neutralization of SARS-CoV-2 Delta virus.

Supplementary Figure 3 Comparison of results from female (f) and male (m) participants younger than 60 years or older than 70 years and fully vaccinated with BNT162b2 regarding T cell response against SARS-CoV-2 Spike, anti-SARS-CoV-2 S IgG titer as well as NT50 values against WT and Delta variant respectively. Medians are visualized as blue (male, m) and red (female, f) lines together with the interquartile range as error bar. Statistical significance between the four groups was determined by Mann-Whitney-U test for nonparametric distribution (*: p<0.05). For individual group sizes of male and female for all comparisons, we refer to **Tables 1** for group >70 y.o. and to **Tables 2** for group <60 y.o. respectively. **(A)** T cell response (IFNγ ELISpots) against SARS-CoV-2 Spike, split up in female and male for both age groups, are presented as Stimulation Index (SI). **(B)** IgG antibody titers against SARS-CoV-2 S domain are shown in binding antibody unit per ml serum (BAU·ml^-1^), separately for female and male of the two age groups. **(C)** Individual titers for 50% neutralization against SARS-CoV-2 WT are shown as dilution factor of serum (1:x). Groups of participants younger than 60 years and older than 70 years respectively are split up in female and male. **(D)** Individual NT50 values against SARS-CoV-2 Delta variant are presented as dilution factor, separately for female and male persons of both age groups. Statistical significance was determined using Mann-Whitney-U test for nonparametric distribution.

Supplementary Table 1 List of medians with corresponding 95% confidence interval (CI) for T cell response, antibody titer and neutralization titer. Medians including lower and upper confidence limit (CL) of T cell response, anti-SARS-CoV-2 S/S1 IgG, NT50 and NT90 values for SARS-CoV-2 WT as well as Delta variant are shown for each participant group.
