# Supplementary figures and images for: Immune Responses Against SARS-CoV-2 WT and Delta Variant in Elderly BNT162b2 Vaccinees
Source: Front Immunol. 2022 Jun 27;13:868361. doi: 10.3389/fimmu.2022.868361 (PMC9271971; doi:10.3389/fimmu.2022.868361)

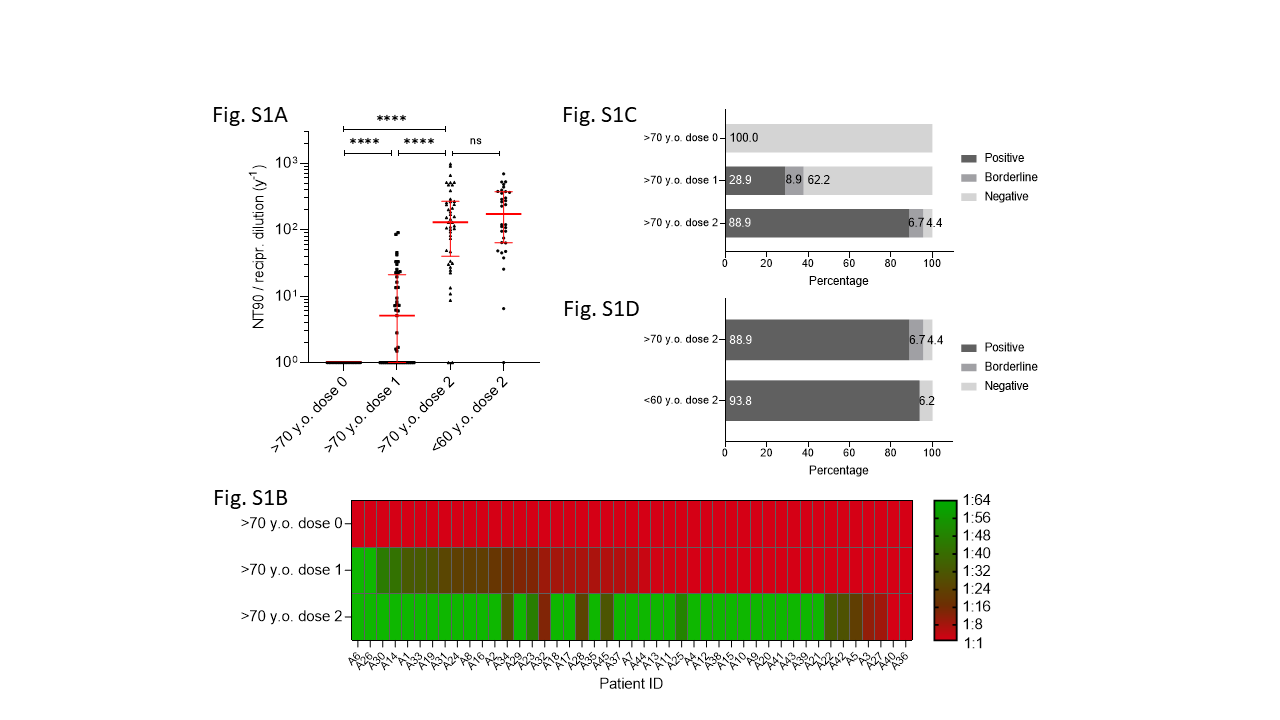

Supplement: Supplementary Figure 1 — Analysis of neutralization titer (NT90) against SARS-CoV-2 WT using sera from participants older than 70 years (n=45) before vaccination (dose 0), after first- (dose 1) and after second vaccination (dose 2) with BNT162b2 in contrast to fully vaccinated participants younger than 60 years (n=32). Neutralization titers are presented as dilution factor for serum (1:x). (A) Individual NT90 values are shown as individual progression for each person until full immunization with BNT162b2. In contrast, individual titers for 90% neutralization are illustrated for people younger than 60 years fully vaccinated with BNT162b2. Medians are visualized es together with the interquartile range as error bar. Statistical significance between values of the three different time points for group >70 y.o. was determined using Wilcoxon test. Mann-Whitney-U test for nonparametric distribution was applied for comparison of immune responses between the two age groups. (B) Heat map for visualization of NT90 progression against SARS-CoV-2 WT before and after vaccination with BNT162b2. Illustrated colors shift from red for a negative- to green for a positive antibody status. (C) Percentages of participants with positive, borderline or negative for 90% neutralization against SARS-CoV-2 WT are shown for time points before-, after first- and after second vaccination. (D) Comparison of percentages of persons older than 70 years and people younger than 60 years with positive, borderline or negative 90% neutralization of SARS-CoV-2 WT virus. [file Image_1.tif]

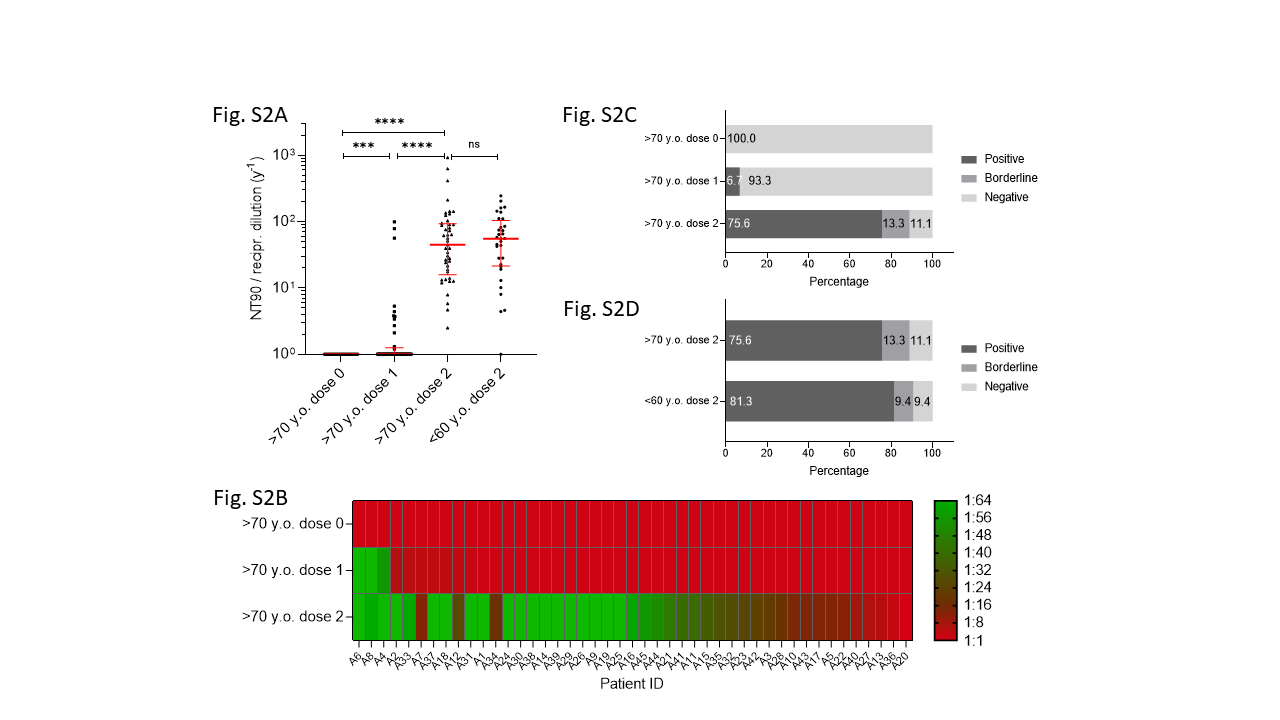

Supplement: Supplementary Figure 2 — Analysis of neutralization titer (NT90) against SARS-CoV-2 Delta variant using sera from participants older than 70 years (n=45) before vaccination (dose 0), after first- (dose 1) and after second vaccination (dose 2) with BNT162b2 in contrast to participants younger than 60 years (n=32) fully vaccinated. Neutralization titers are presented as dilution factor for serum (1:x). (A) Individual NT90 values are shown as individual progression for each person until full immunization with BNT162b2. In contrast, individual titers for 90% neutralization are illustrated for people younger than 60 years fully vaccinated with BNT162b2. Medians are visualized together with the interquartile range as error bar. Statistical significance between values of the three different time points for group >70 y.o. was determined using Wilcoxon test. Mann-Whitney-U test for nonparametric distribution was applied for comparison of immune responses between the two age groups. (B) Heat map for visualization of NT90 progression against SARS-CoV-2 Delta variant before and after vaccination with BNT162b2. Illustrated colors shift from red for a negative- to green for a positive antibody status. (C) Percentages of participants with positive, borderline or negative for 90% neutralization against SARS-CoV-2 Delta variant are shown for time points before-, after first- and after second vaccination. (D) Comparison of percentages of persons older than 70 years and people younger than 60 years with positive, borderline or negative 90% neutralization of SARS-CoV-2 Delta virus. [file Image_2.tif]

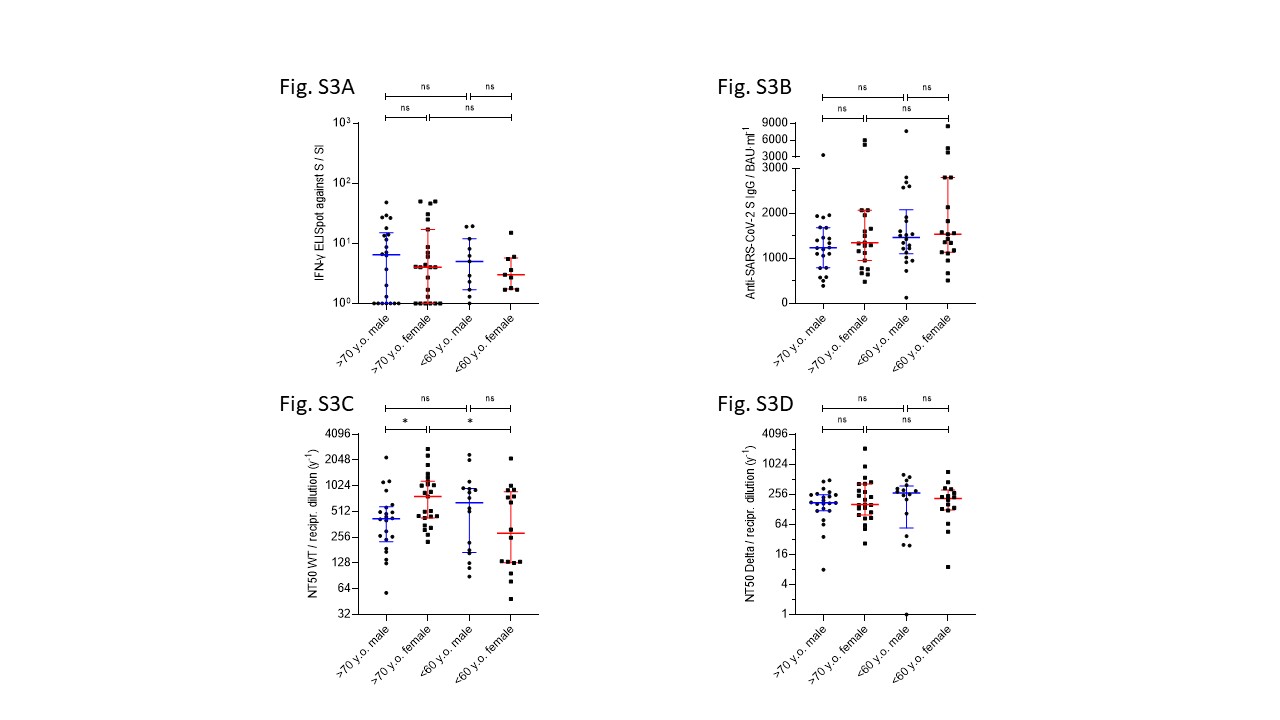

Supplement: Supplementary Figure 3 — Comparison of results from female (f) and male (m) participants younger than 60 years or older than 70 years and fully vaccinated with BNT162b2 regarding T cell response against SARS-CoV-2 Spike, anti-SARS-CoV-2 S IgG titer as well as NT50 values against WT and Delta variant respectively. Medians are visualized as blue (male, m) and red (female, f) lines together with the interquartile range as error bar. Statistical significance between the four groups was determined by Mann-Whitney-U test for nonparametric distribution (*: p<0.05). For individual group sizes of male and female for all comparisons, we refer to Tables 1 for group >70 y.o. and to Tables 2 for group <60 y.o. respectively. (A) T cell response (IFNγ ELISpots) against SARS-CoV-2 Spike, split up in female and male for both age groups, are presented as Stimulation Index (SI). (B) IgG antibody titers against SARS-CoV-2 S domain are shown in binding antibody unit per ml serum (BAU·ml-1), separately for female and male of the two age groups. (C) Individual titers for 50% neutralization against SARS-CoV-2 WT are shown as dilution factor of serum (1:x). Groups of participants younger than 60 years and older than 70 years respectively are split up in female and male. (D) Individual NT50 values against SARS-CoV-2 Delta variant are presented as dilution factor, separately for female and male persons of both age groups. Statistical significance was determined using Mann-Whitney-U test for nonparametric distribution. [file Image_3.tif]
