# Supplementary material for: Immune Responses Against SARS-CoV-2 WT and Delta Variant in Elderly BNT162b2 Vaccinees
Source: Front Immunol. 2022 Jun 27;13:868361. doi: 10.3389/fimmu.2022.868361 (PMC9271971; doi:10.3389/fimmu.2022.868361)
Supplement: Supplementary Table 1 — List of medians with corresponding 95% confidence interval (CI) for T cell response, antibody titer and neutralization titer. Medians including lower and upper confidence limit (CL) of T cell response, anti-SARS-CoV-2 S/S1 IgG, NT50 and NT90 values for SARS-CoV-2 WT as well as Delta variant are shown for each participant group. [file Table_1.pdf]

Tab. S1

**Participant group for IFN-γ T-cell response**	**SI (lower CL-upper CL at 95%)**
>70 y.o. dose 0	1.0 (95% CI 1.0 - 1.0)
>70 y.o. dose 1	1.5 (95% CI 1.0 - 2.0)
>70 y.o. dose 2	4.3 (95% CI 1.7 - 8.7)
<60 y.o. dose 2	3.3 (95% CI 1.8 - 6.2)

**Participant group for IgG antibody test**	**Median antibody titer (lower CL-upper CL at 95%)**
>70 y.o. dose 0	4.8 (95% CI 4.8 - 4.8)
>70 y.o. dose 1	142.0 (95% CI 107.0 - 260.0)
>70 y.o. dose 2	1330.0 (95% CI 1120.0 - 1600.0)
<60 y.o. dose 2	1512.0 (95% CI 1291.0 - 1827.0)

**Participant group and virus**	**Median NT50 (lower CL-upper CL at 95%)**	**Median NT90 (lower CL-upper CL at 95%)**
>70 y.o. dose 0 WT	1.0 (95% CI 1.0 - 1.0)	1.0 (95% CI 1.0 - 1.0)
>70 y.o. dose 1 WT	68.1 (95% CI 37.8 - 119.6)	5.1 (95% CI 1.0 - 9.4)
>70 y.o. dose 2 WT	481.8 (95% CI 415.7 - 768.0)	129.8 (95% CI 93.3 - 211.9)
<60 y.o. dose 2 WT	536.0 (95% CI 134.3 - 909.4)	173.6 (95% CI 75.1 - 356.3)
>70 y.o. dose 0 Delta	1.0 (95% CI 1.0 - 1.0)	1.0 (95% CI 1.0 - 1.0)
>70 y.o. dose 1 Delta	1.0 (95% CI 1.0 - 12.3)	1.0 (95% CI 1.0 - 1.0)
>70 y.o. dose 2 Delta	172.6 (95% CI 137.2 - 240.5)	44.7 (95% CI 25.4 - 75.6)
<60 y.o. dose 2 Delta	231.5 (95% CI 131.1 - 309.9)	55.5 (95% CI 28.1 - 81.9)
